# Nitrogen Gas-Assisted Extrusion for Improving the Physical Quality of Pea Protein-Enriched Corn Puffs with a Wide Range of Protein Contents

**DOI:** 10.3390/foods13152411

**Published:** 2024-07-30

**Authors:** Siwen Luo, Jitendra Paliwal, Filiz Koksel

**Affiliations:** 1 Department of Food and Human Nutritional Sciences, University of Manitoba, Richardson Centre for Food Technology and Research, 196 Innovation Drive, Winnipeg, MB R3T 2N2, Canada; luos345@myumanitoba.ca; 2 Department of Biosystems Engineering, University of Manitoba, E2-376, EITC, 75A Chancellor’s Circle, Winnipeg, MB R3T 2N2, Canada; j.paliwal@umanitoba.ca

**Keywords:** high-protein snack, physical blowing agent, expansion, extrudate density, hardness, crispness, crunchiness, texture, Young’s modulus, mechanical properties

## Abstract

Blowing agent-assisted extrusion cooking is a novel processing technique that can alter the expansion of extruded snacks and, thus, enhance their physical appeal, such as texture. However, to this day, this technique has only been studied for ingredients with limited protein contents (<30%). In this study, protein-enriched snacks were extruded using nitrogen gas as a blowing agent at a wide protein range (0–50%) to better explore the potential of this technique in manufacturing high-protein snacks. The results showed that, with nitrogen gas injection, extrudate radial expansion was significantly (*p* < 0.05) improved at 10% and 40% protein, while extrudate density was significantly reduced at 30% and 50% protein. Nitrogen gas-injected extrudates, especially at 50% protein, exhibited improvements in texture, including a reduction in hardness and an increase in crispness. Collectively, this study showed the promising potential of nitrogen gas-assisted extrusion in improving the physical appeal of innovative healthy snacks at a high protein level (i.e., 50%).

## 1. Introduction

Snacks are an essential component of daily nutrition intake. However, excessive snacking, particularly on unhealthy options, namely those high in lipids and rapidly digestible carbohydrates, can contribute to metabolic disorders [1]. In response to this concern, food manufacturers are challenged to incorporate more nutritious ingredients in snack formulations without compromising physical appeal, such as lightness, crispness, and crunchiness. The global market for healthy snacks was estimated at USD 90.62 billion in 2022, with projections of a remarkable compound annual growth rate of 6.7% from 2023 to 2030 [2].

In line with these trends, many studies have attempted to use protein-enriched formulas in extruded snacks, as an increase in protein intake is proven to have several health benefits, such as helping muscle synthesis and fat loss [3]. Most attempts at raising the protein content of snack foods have resulted in impairing the snacks’ sensory appeal, causing them to become denser and harder [4,5,6]. A desirable extruded puffed snack exhibits characteristics such as high expansion, low density, low hardness, high crispness, and high crunchiness [7]. These undesirable alterations in product characteristics result from changes in expansion dynamics (i.e., bubble growth and shrinkage) caused by protein addition, which negatively affect the sensory qualities of snack foods [6], undermining the goal of offering more appealing and nutritious food options in the market to promote increased protein consumption. Gas injection-assisted extrusion cooking is one way to overcome these challenges [7].

Gas injection-assisted extrusion combines conventional extrusion cooking with an injection of different gases (e.g., nitrogen, carbon dioxide, air, etc.) as additional blowing agents to water vapor. During extrusion processing, depending on the choice of gas (e.g., nitrogen), additional nucleation sites in the melt can be created, driving the cells in the food matrix to grow and expand [8,9] and thus potentially enhancing product quality [9,10,11]. Compared to other gases used in extrusion cooking, nitrogen gas has the advantage of being inert, e.g., when compared to CO_2_, and non-flammable, e.g., when compared to O_2_ [7]. However, the effects of nitrogen gas in such food systems are highly dependent on its concentration and the way it interacts with different food ingredients of varying thermal and rheological properties during the extrusion cooking process. Furthermore, the existing research is limited to pulse/cereal flour-snack products (e.g., those made from yellow pea and red lentil flours), with relatively restricted protein content ranging from 0 to ~25% [7,9,11]. Thus, the potential of nitrogen gas-assisted extrusion cooking has remained relatively untapped in the context of high-protein snacks with protein levels comparable to other high-protein snacks on the market, e.g., high-protein snack bars or jerky-type products that contain >35% protein [7]. Moreover, a systematic investigation of how nitrogen gas acts in high-protein food systems during extrusion is still needed, as a comprehensive understanding of this process can potentially help food manufacturers manipulate and optimize end-product properties of high-protein puffed snacks and breakfast cereals [7].

To bridge this research gap, the objective of this study was to investigate the effects of nitrogen gas injection at different pressures on the physical (i.e., radial expansion index and extrudate density), textural (i.e., hardness, crispness, and crunchiness) and mechanical (i.e., Young’s modulus, flexural stress, and fracture strain) properties of pea protein-fortified extruded snacks made with a wide range of protein content (0–50%). The correlations among protein content, extrusion parameters (e.g., nitrogen injection pressure, motor torque, and die pressure), and the end product properties (e.g., expansion, density, and texture) are also investigated to provide some fundamental information on how these factors interact in a complex food extrusion system.

## 2. Materials and Methods

### 2.1. Materials

Commercial corn starch was purchased from Cargill Inc. (Minnetonka, MN, USA). Commercial pea protein isolate (FYPP-80-B) was provided by AGT Food and Ingredients Inc. (Regina, SK, Canada). The protein, ash, and lipid contents of the corn starch and the pea protein isolate (PPI) were measured using AACC International Methods 08-01.01, 46-13.01, and 30-25.01, respectively. For extrusion, the corn starch and PPI were blended to reach six different protein levels (0, 10, 20, 30, 40, and 50% protein on a dry basis). The blends were initially prepared in 1 kg batches using a KitchenAid^®^ mixer (Benton Charter Township, MI, USA) at low speed for 15 min and then transferred to a Hobart mixer (Hobart Cooperation, Troy, NY, USA) for further mixing at low speed for 10 min in 4 kg batches. After mixing, the protein content of different corn starch-pea protein blends was measured again to ensure an even and accurate mixing ratio. Commercial flow conditioning silica (FLOW-GARD^TM^ FF) was supplied by PPG Industries Inc. (Monroeville, PA, USA) and added to improve the flowability of the corn starch-pea protein blends at a ratio of 1.5 g per 100 g dry blend.

### 2.2. Particle Size Analysis

The particle size distributions of the raw materials were determined by Mastersizer 3000 (Malvern Instruments Ltd., Malvern, UK) and its control software, using a refractive index of 1.460 for corn starch and pea protein, a refractive index of 1.480 for silica and an absorption factor of 0.01 for all the particles tested.

### 2.3. Extrusion Process

A lab-scale co-rotating twin-screw extruder (MPF19, APV Baker Ltd., Peterborough, UK) with a 25:1 screw length-to-diameter ratio was used for extrusion cooking. The barrel temperature profile of the five temperature-controlled zones of the extruder barrel was set at 75, 95, 115, 130, and 145 °C, respectively, from the feed entrance toward the extruder die exit for all extrusion runs. The barrel screw configuration following the configuration reported by Koksel and Masatcioglu [11] and a screw speed of 300 rpm were kept constant. A circular die with a diameter of 5 mm and a length of 19.35 mm was used. Before extrusion, the feed rate was calibrated to 2.2 kg dry material per hour, and the distilled water injection rate was calibrated to reach a feed moisture content of 30% on dry material basis. These extrusion conditions were selected based on preliminary experiments, ensuring compatibility with the capacity of the extruder and providing acceptable extrudate expansion across the wide range of protein levels and nitrogen gas injection pressures examined. Furthermore, to isolate the influence of protein content and nitrogen gas injection pressure and to eliminate confounding effects from varying extrusion parameters, constant screw configuration and speed, barrel temperature profile, dry feed rate, and feed moisture content were employed. During extrusion cooking, two nitrogen gas injection pressures (150 and 300 kPa) were achieved by injecting nitrogen gas from a nitrogen t-tank (Innovair Group, Winnipeg, MB, Canada) through a gas pressure regulator located 226 mm away from the die exit. Conventional extrusion (i.e., without nitrogen gas injection) was performed as a control.

Extrusion experiments were designed as a full factorial (6 feed formulas × 3 nitrogen gas injection pressures = 18 treatments), with each treatment extruded in triplicates. A full factorial design was chosen to systematically explore all possible combinations of factors and levels, thereby providing a more comprehensive understanding of the main effects and their interactions. During extrusion cooking, motor torque (abbreviated as torque) and die pressure were recorded in quadruplicates. The specific mechanical energy (SME) for each extrusion run was calculated using Equation (1) [9]:

(1) $$SME=\frac{Real\;screw\;speed}{Maximum\;screw\;speed}\times \frac{Torque}{100}\times \frac{Extruder\;motor\;powder}{Total\;mass\;flow\;rate}$$

where the maximum screw speed was 500 rpm, and the extruder motor power was 2.2 kW. The SME values were presented as the means of the triplicated extrusion runs. After extrusion, extrudates were dried overnight in an air oven at 40 °C and stored in sealed plastic bags for further analyses.

### 2.4. Radial Expansion Index, Extrudate Density and Porosity

The radial expansion index was reported as the ratio of the extrudate diameter to the die diameter (i.e., 5 mm). The diameter of extrudates was measured using a digital caliper (Control Company, Friendswood, TX, USA). For each extrusion run, 15 measurements were taken from 4 randomly selected extrudate pieces. The results were presented as the average of the triplicated extrusion runs.

The extrudate density was measured using a mass displacement method using canola seeds, as reported by Koksel and Masatcioglu [11]. Approximately 5 g of 5 cm-long extrudate pieces were used for each measurement. For each extrusion run, five measurements were taken, and the results were presented as the average of the triplicated extrusion runs.

The extrudate porosity (∅) was calculated using Equation (2) [9]:

(2) $$\emptyset \left(\%\right)=\left(1-\frac{\rho_{extrudate}}{\rho_{cell\;wall}}\right)\times 100\%$$

where, $\rho_{extrudate}$ represents the extrudate density in g/cm^3^, measured as described above, and $\rho_{cell\;wall}$ represents the density of the extrudate cell walls in g/cm^3^. To measure ${the\;\rho }_{cell\;wall}$, extrudates were ground into powder using an ultracentrifugal mill (Retsch, ZM 200, Haan, Germany) equipped with a 0.25 mm sieve. The density of the ground extrudate was measured using a gas displacement pycnometer (Ultrapyc 1200e, Quantachrome Instruments, Boynton Beach, FL, USA) following the method reported by Luo et al. [9]. For each sample, approximately 3.5 g of ground extrudate was weighed into a sample chamber, sealed, and purged with helium gas at a pressure of 131 kPa. The cell wall density was measured in triplicates for each ground extrudate sample, and the porosity was calculated based on the mean values of the triplicated extrusion runs for each extrusion treatment.

### 2.5. Texture and Mechanical Properties

A Texture analyzer (TA-XT-plus, Stable Micro Systems, Godalming, UK) equipped with a 5 kg load cell was used to perform the texture analysis. Randomly selected extrudate samples were placed in an air oven at 40 °C for 24 h to reach an equilibrium moisture of approximately 6–8% (wet basis) prior to the analysis. A cutting test was performed using a 1 mm-thick Warner–Bratzler shear blade probe on 4 cm long extrudates and following the method reported by Koksel and Masatcioglu [11]. Three-point bending test was performed using a three-point bend rig (HDP/3PB, Stable Micro Systems, Godalming, UK) following the method reported by Robin et al. [12]. Before the analysis, the diameter of the extrudate was measured using a digital caliper and recorded. The compatible software, Exponent Connect (version 6,1,16,0, Stable Micro Systems, Godalming, UK), was used to extract extrudate hardness, crispness, and crunchiness from the cutting test, maximum force (f) and probe displacement (δ) at fracture from the bending test. The normalized crispness (or crunchiness) of an extrudate was calculated by dividing the crispness (or crunchiness) by the diameter of that extrudate sample. The Young’s modulus (E), flexural stress (σ), and fracture strain (ε) were calculated using Equations (3)–(5) [13,14]:

(3) $$E=\frac{4fL^{3}}{3\pi \delta D^{4}}$$

(4) $$\sigma =\frac{24fL}{\pi D^{3}}$$

(5) $$\varepsilon =\frac{\sigma }{E}$$

where L and D represent the length between two supports (i.e., 50 mm) and the extrudate diameter, respectively. The textural attributes were reported as the mean of three measurements, each of which was the mean of readings from five random extrudates for the cutting test and the mean of five measurements, each of which was the reading from one random extrudate for the bending test. The results were presented as the average of the triplicated extrusion runs.

### 2.6. Statistical Analysis

The effects of protein content and nitrogen gas injection pressure on extrudate properties were analyzed using a two-way analysis of variance (ANOVA). Statistical differences in the physical properties of extrudates were determined using Tukey’s test (*p* < 0.05). Tukey’s test was selected for its conservativity. Pearson’s correlation coefficients were determined to assess the relationship between processing conditions and extrudate properties. All statistical analyses were performed using OriginPro, version 2023 (OriginLab Corporation, Northampton, MA, USA). After ANOVA, the normality and homogeneity of variance were confirmed using SAS^®^ Studio (© 2024 SAS Institute Inc., Cary, NC, USA).

## 3. Results and Discussion

### 3.1. Proximate Composition and the Particle Size Distribution of the Raw Materials

The protein, ash, and lipid contents of the corn starch were 0.12 ± 0.07%, 0.07 ± 0.01%, and 0.05 ± 0.04% (dry basis), respectively. The protein, ash, and lipid contents of the pea protein isolate were 78.60 ± 3.80%, 8.92 ± 0.75%, and 0.30 ± 0.15% (dry basis), respectively.

**Figure 1:**
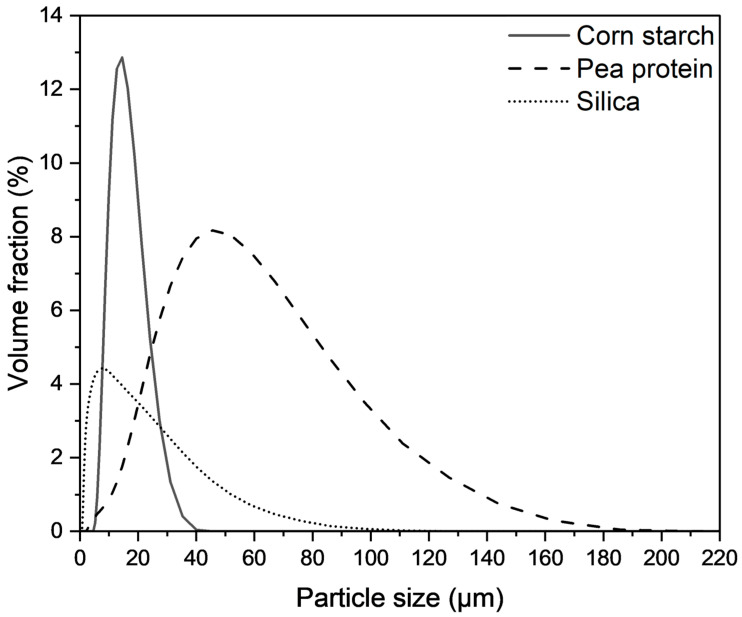
Particle size distributions of the raw materials.

The particle size distributions and the key particle features of the raw materials are presented in Figure 1. The corn starch had particles varying from 5.2 to 40.1 μm in size, while the pea protein isolate had a wider particle size distribution, varying from 2.4 to 186 μm. It is essential to report the particle size of ingredients in food extrusion studies because determining the optimal fineness or coarseness of powder ingredients for different end-use applications is necessary to ensure consistent outcomes, especially for pulse-based ingredients like pea protein where industry standards do not exist [15].

### 3.2. Extrusion Parameters

Extrusion parameters, including torque, die pressure, and specific mechanical energy (SME) input, are presented in Table 1. Two-way ANOVA (results presented in Table 2) showed that the protein content and nitrogen gas injection pressure both had significant effects on die pressure, torque, and specific mechanical energy (*p* < 0.0001). The interaction between protein content and nitrogen gas injection pressure on these parameters was also significant (*p* < 0.0001).

For conventional (i.e., without gas injection) extrusion, increasing the incorporation level of pea protein into corn starch from 10 to 30% significantly lowered the die pressure. At 40% protein, the die pressure increased to a level comparable to the 0% protein formula, while at 50% protein, the die pressure increased further to reach its highest level. The highest die pressure at 50% protein could be resulting from changes in the melt matrix with protein addition, i.e., phase inversion from a continuous starch phase with dispersed proteins to co-continuous phases of starch and proteins or a continuous protein phase with a dispersed starch phase [16,17]. For conventional extrusion cooking, torque and die pressure had a significant and strong positive correlation (r = 0.99, *p* < 0.001, presented in Table 3), indicating that a higher melt viscosity, manifested by higher torque, generated higher die pressure in conventional extrusion. However, a negative correlation between torque and die pressure (r = −0.56, *p* < 0.05, presented in Table 4) was found when nitrogen gas-assisted extrusion was also considered.

In most cases, injecting nitrogen gas caused significant (*p* < 0.05) die-pressure reduction during extrusion, for example, comparing 0 and 150 kPa gas injection conditions for the 10% protein formula. Some exceptions to this significant die pressure drop were the 0 and 50% protein formulas at 150 kPa gas injection and the 30% protein formula at 150 and 300 kPa gas injection, where the decline was statistically insignificant. Similar reductions in die pressure were also reported for extrusion with supercritical CO_2_ [8,19], where the die pressure drop was explained by the melt viscosity-reduction effect of supercritical CO_2_. However, a viscosity-reduction effect is not expected due to the relatively lower solubility of N_2_ compared to CO_2_ and was also not observed, as evidenced by the increase in torque values with an increase in nitrogen gas injection pressure (Table 1). Our results indicate that the introduction of nitrogen gas changed the flow characteristics of the melt at the die exit. Further studies on the melt rheological properties are needed to better explain the change in die pressure caused by nitrogen gas injection.

The torque values during extrusion represent the resistance experienced by the extruder motor and depend on several factors, including the degree of extruder barrel fill, melt viscosity, and screw speed [11,20]. The torque value was the lowest for the 50% protein formula among all the conventional extrusion runs. Proteins can also have a lubricating effect that reduces torque [21]. In addition, studies have indicated that materials with larger particle sizes, such as pea protein isolate in this study, experience a lower contact with the extruder barrel during the extrusion process, due to their lower surface area to volume ratio. Consequently, they are not heated to as high temperatures compared to ingredients with relatively lower particle sizes and exhibit higher melt viscosity [22]. This may have contributed to the higher die pressure value observed at the highest protein content studied (i.e., 50% protein). Overall, nitrogen gas injection either did not change or increase the torque values during extrusion compared to those of 0% protein formula, with the exception of 150 kPa nitrogen gas injection at 30% protein. It is possible that the increase in torque brought about by nitrogen gas injection was due to the increase in barrel fill with the additional gas volume [20]. The specific mechanical energy input had the same trend as torque values.

### 3.3. Radial Expansion Index, Extrudate Density and Porosity

**Figure 2:**
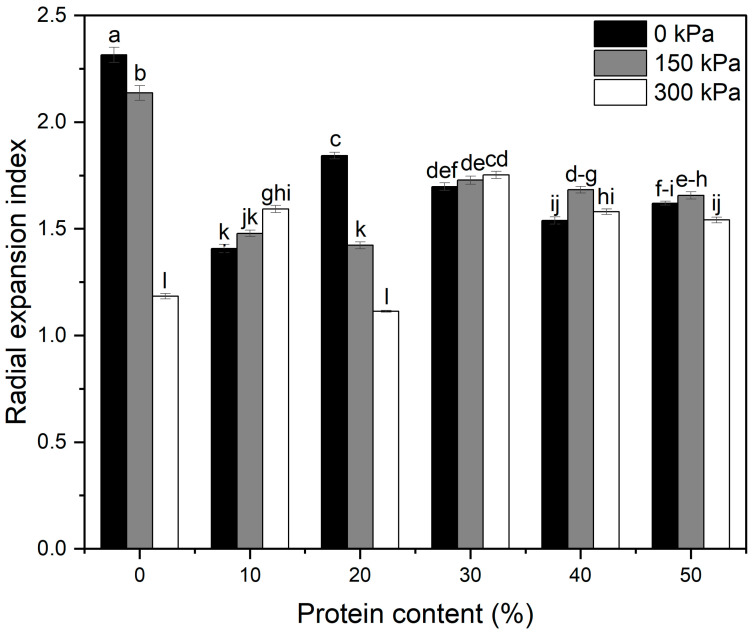
Effects of protein content and nitrogen gas injection on radial expansion index. Error bars represent ± standard errors of means (*n* = 45). Different letters represent statistically significant differences among treatments (*p* < 0.05).

The results of the radial expansion index (i.e., the degree of expansion in the direction parallel to the die plate) are presented in Figure 2. The digital image of extrudates at varying protein contents and nitrogen gas injection pressures is provided in the Supplementary Material (Figure S1). Statistical analysis showed that protein content and nitrogen gas injection pressure significantly affected extrudate radial expansion (*p* < 0.0001). The two-way interaction between protein content and nitrogen gas injection pressure on the radial expansion index was also significant (*p* < 0.0001).

For the samples with no gas injection, the 0% protein formula (i.e., corn starch) had the highest radial expansion, and the addition of protein significantly (*p* < 0.05) reduced extrudate radial expansion. Consistent with our findings, Roudaut et al. [23] also reported that extrudates made with pure corn starch had the greatest expansion when compared to whey protein-supplemented corn starch extrudates. The increase in protein content from 20 to 40% caused a significant (*p* < 0.05) decrease in radial expansion. Phillipp et al. [5] reported that pea protein isolates at 30–50% levels reduced the radial expansion significantly by causing a greater extent of shrinkage during melt expansion. Pea protein addition has been shown to reduce the glass transition temperature of the melt [24], and, therefore, it can delay melting solidification, permitting a longer time for the melt to shrink before the extrudate solidified.

Moreover, proteins suppress the extensibility of starch by forming a rigid network that decreases the mobility of amylopectin chains, resulting in higher resistance to melt expansion [17,25]. Thus, adding proteins to the formula generally reduces the expansion of extruded puffed products. Interestingly, the radial expansion at 20% protein was higher than at 10% when conventionally extruded. The positive effect of protein up to 20% on expansion might result from its thermosetting effect [26]. Once thermosetting materials (e.g., proteins) are heated over their glass transition temperature and cooled down, they form a rigid gel to set the extrudate structure and thus inhibit any further shrinkage [27]. At protein contents higher than 20%, it is possible that protein’s suppression on starch extensibility outweighed the positive thermosetting effect on extrudate radial expansion, which in turn may have caused the aforementioned reductions in radial expansion.

The effect of nitrogen gas injection pressure on extrudate radial expansion was a function of protein content, as evident from the significant interaction between protein content and nitrogen gas injection. Significant improvement in radial expansion was observed at 150 and 300 kPa at 10% protein, while the improvement at 40% protein was only observed at 150 kPa. The same improvements in radial expansion were also reported for red lentil flour extrudate produced with 300 kPa nitrogen gas injection [28], as well as for wheat flour extrudates at 100 and 200 kPa nitrogen gas injection [29]. When the melt leaves the extruder at the die exit in conventional extrusion, water evaporation due to the fast pressure drop becomes the driving force for product expansion [30]. When additional gas (e.g., nitrogen gas) is introduced into the extruder barrel, it may favor bubble nucleation and expansion after the melt exits the die, thus producing more expanded products [8,31]. In contrast, nitrogen gas injection at 150 and 300 kPa reduced the radial expansion significantly (*p* < 0.05) at 0 and 20% protein. Similar reductions in the radial expansion were reported for red lentil flour-based extrudates produced at 300, 400, and 500 kPa nitrogen gas injection [9,10] and yellow pea flour extrudates with 300, 400, and 500 kPa nitrogen gas injection [11]. It has been reported that different extrudate matrices have different capacities to hold gas bubbles trapped inside the melt [32] and that the impaired gas-holding properties of starch melts may lead to the gas being lost to the atmosphere and thereafter cause structural collapse and lower overall expansion [9]. In addition, the die pressure is positively related to the solubility of nitrogen gas in the melt, according to Henry’s law [33,34]. Therefore, the cause of the less expanded products could be the relatively lower solubility of nitrogen gas at reduced die pressure. From another perspective, the thermosetting proteins in the melt would impart rigidity to extrudates, which may strain both extrudate expansion and shrinkage [27]. Our results indicated that these confounding factors contributed to the different responses in extrudate radial expansion at different protein levels.

**Figure 3:**
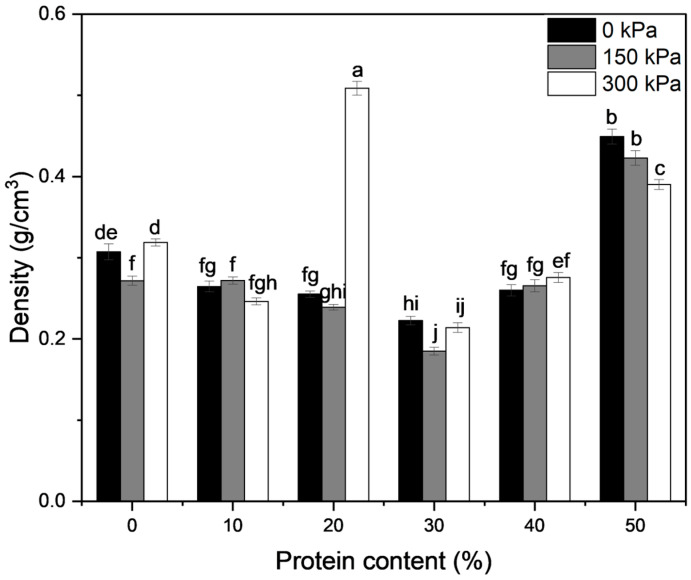
Effects of protein content and nitrogen gas injection on extrudate density. Error bars represent ± standard error of mean (*n* = 15). Different letters represent statistically significant differences among treatments (*p* < 0.05).

**Figure 4:**
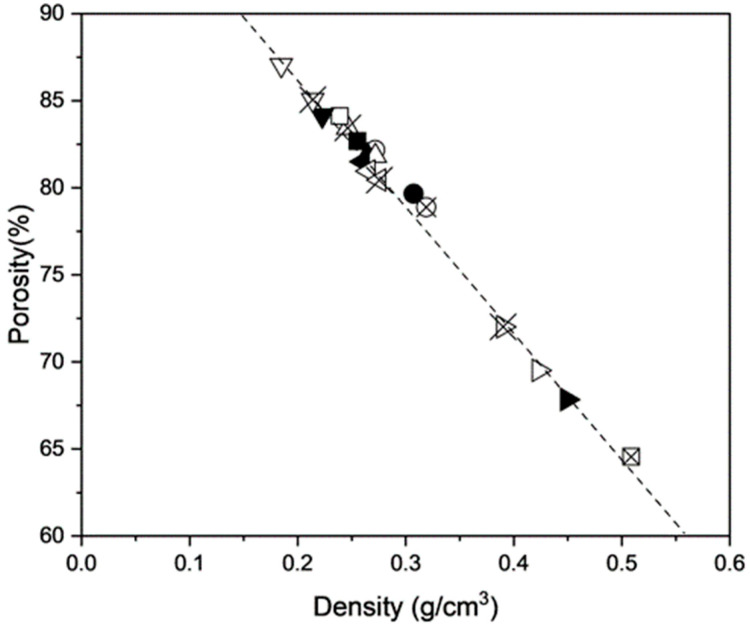
Extrudate porosity (∅) and density (ρ) as a function of protein content (●: 0%, ▲: 10%, ■: 20%, ▼: 30%, ◀: 40%, and ▶: 50%, d.b.) and different nitrogen gas injection pressure (solid shape: 0 kPa, hollow shape: 150 kPa, and crossed shape: 300 kPa). The dashed line represents the linear trendline with the equation ∅=−72.64 ρ+101.

Extrudate density as a function of protein content and nitrogen gas injection pressure is presented in Figure 3. According to two-way ANOVA results, both protein content and nitrogen gas injection pressure had significant effects on extrudate density (*p* < 0.0001), and the interactive effect between protein content and nitrogen gas injection pressure on extrudate density was also significant (*p* < 0.0001). When comparing the extrudate radial expansion index with extrudate density, it can be seen that the extrudate density was not perfectly negatively correlated to the radial expansion index, which was also reported by Chan et al. [10]. Instead., a significant and strong negative correlation (r = −0.99, *p* < 0.001) was observed between porosity and density, as depicted in Figure 4. This is interesting because there have also been reports of an inverse relationship between the two expansion ratios [25,35]. This finding suggests that the radial expansion index alone is inadequate to describe extrudate expansion, as expansion also happens in the longitudinal direction (i.e., the direction parallel to the flow direction of extrudate through the die) [36]. The 50% protein formula had the highest density for extrudates produced with no gas, while the 30% protein formula had the lowest. This is in agreement with the study that reported a decrease in extrudate gas volume fraction (i.e., an increase in density) when soy protein content was raised from 20 to 50%, probably due to the reduced melt extensibility and enhanced melt rigidity that was previously mentioned [17]. Strong correlations found between density and torque (r = 0.96, *p* < 0.01), as well as density and die pressure (r = 0.98, *p* < 0.001), also imply that the high extrudate density is possibly a result of high melt viscosity that restricted the overall extrudate expansion [7].

Similar to the radial expansion index, the effect of nitrogen gas injection pressure on density was also a function of protein content. Nitrogen gas injection at 300 kPa caused profound densification of the 20% protein extrudates. This is in agreement with the results obtained for nitrogen gas assisted-extrusion of red lentil and yellow pea flours [9,11]. Pea protein at a 20% level may have weakened the gas-holding properties and reduced the glass transition temperature of the melt while not imparting as much rigidity to the melt as it would at higher protein levels (i.e., 30–50%). On the contrary, nitrogen gas injection pressure significantly (*p* < 0.05) reduced the extrudate density at 0 and 30% protein at 150 kPa injection and 50% protein at 300 kPa injection. The decreases in density suggest a higher degree of overall expansion, which was also reported for extruded yellow pea flour [11], red lentils [10], and wheat flour [10,11,29]. When comparing the 0 and 20% protein extrudates at 300 kPa nitrogen gas injection, it was interesting to see that despite having similar radial expansions, the 20% protein sample had a much higher density, suggesting that it was much less expanded in the longitudinal direction. Nitrogen gas injection did not significantly affect extrudate density at 40% protein at the two pressures studied.

### 3.4. Texture and Mechanical Properties

The most critical texture attributes of cellular snack foods, namely hardness, crispness, and crunchiness [37], are presented in Table 5. The crispness and crunchiness were normalized using the diameter of the extrudates to remove the effect of radial extrudate expansion on these two properties. In addition, Young’s modulus, flexural stress, and fracture strain were determined through a bending test to characterize the extrudates’ mechanical properties, providing a comprehensive understanding of the extrudates’ structural properties and performance under stress.

In terms of hardness, both protein content (*p* < 0.0001) and nitrogen gas injection pressure (*p* < 0.001) had significant effects on extrudate hardness. The interaction between these two factors was also significant (*p* < 0.0001). For the treatments without gas injection, extrudates with 0 and 50% protein content had the highest hardness. Similar results were also reported, showing that adding pea protein isolate at 30–50% reduced rice starch-based extrudates’ breaking force [5]. In contrast, the incorporation of soybean protein concentrate up to 40% dramatically lowered the hardness of cornmeal flour-based extrudates [38]. It is commonly recognized that more expanded products have lower peak breaking stress [26]. Contrary to the literature, a strong positive correlation between radial expansion and hardness (r = 0.90, *p* < 0.05) was found for conventionally extruded samples in this study, and the most expanded treatment in this study (0% protein with no gas) turned out to have the highest peak force (i.e., hardness). This result indicated that the relatively higher hardness for this treatment was mostly contributed by the mechanical properties of the solid phase (i.e., the cell walls) rather than a lack of expansion. It should be noted that the compositional homogeneity of the solid phase at a small localized scale impacts the fracture behavior of cellular products [39,40]. The weakening of the mechanical properties of this matrix due to protein disruption has been reported in various studies [16,17,41,42]. A starch matrix can lose its continuity under thermo-mechanical treatment during extrusion cooking when protein aggregates, which are incompatible with the starch, are introduced into the matrix [16,17,43]. As the protein level increases further, more protein aggregates form. When protein content reaches a certain level, the continuous phase of the melt switches from starch to protein, which may harden the cell walls. This cell wall hardening effect of protein aggregates, combined with the less expanded structure at 50% protein, possibly led to higher extrudate hardness than lower protein-containing extrudates or produced by conventional extrusion cooking.

In terms of the effect of nitrogen gas injection at 0% protein content, 150 kPa gas injection caused a slight reduction in extrudate hardness. Extrudate hardness did not further decrease with the injection pressure increasing to 300 kPa. At 20% protein, 150 kPa gas injection caused a significant reduction in extrudate hardness compared to no gas injection. However, extrudate hardness at 300 kPa increased and was comparable to the extrudate with no gas injection. Significant effects of protein content (*p* < 0.0001) and nitrogen gas injection pressure (*p* < 0.001) on extrudate crunchiness were found. The interaction effect between protein content and nitrogen gas injection on extrudate crispness was also significant (*p* < 0.0001). However, when crispness was normalized, the effect of nitrogen gas injection was no longer significant.

For the samples with no gas injection, extrudates with 50% protein had the lowest crispness and normalized crispness, which is in line with the results of Philipp et al. [5], who reported that extrudates with 45% pea protein isolate concentration had only a few fracture events. This low crispness could be attributed to the increased extrudate density due to the presence of pea protein at a 50% level, as extrudate density was strongly and negatively correlated to crispness (r = −0.83, *p* < 0.001). A similar correlation between crispness and extrudate density was also reported by Barrett et al. [44] for corn meal extrudates. With 300 kPa gas injection, all extrudates had similar or significant (*p* < 0.05) lower crispness values when compared to their no-gas counterparts. This trend agrees with what was reported for yellow pea flour extrudates (protein content ~24%, w.b.). Among the extrudates with 300 kPa gas injection, the lowest crispness was observed for those with 0 and 20% protein.

For crunchiness, protein content, and nitrogen gas injection pressure significantly affected extrudate crunchiness (*p* < 0.0001). The interaction between protein content and nitrogen gas injection pressure on extrudate crunchiness was also significant (*p* < 0.0001). Protein content and nitrogen gas injection significantly affected the normalized crunchiness (*p* < 0.0001 and *p* < 0.05), while their interaction was insignificant.

Extrudates with 0% protein and no gas injection had the highest crunchiness and normalized crunchiness. At 0% protein, 150 kPa gas injection significantly reduced crunchiness, and an increase in gas pressure to 300 kPa caused a further reduction in crunchiness. At 20% protein, 150 kPa gas injection significantly reduced crunchiness, but a further increase in pressure from 150 kPa to 300 kPa did not cause any significant changes. Extrudates with 0% protein had the highest normalized crunchiness, while extrudates with 10–40% protein had similar normalized crunchiness. Pearson’s correlation coefficients showed that crunchiness was strongly and positively correlated to radial expansion (r = 0.88, *p* < 0.0001) and hardness (r = 0.83, *p* < 0.0001). In contrast, the normalized crunchiness was only strongly and positively correlated to hardness (r = 0.90, *p* < 0.0001). This is in line with the results of Van Vilet and Primo-Martin [37], reporting that a certain level of hardness is needed in foods to be perceived as crunchy.

Similar to crunchiness, protein content, and nitrogen gas injection pressure significantly affected extrudates’ mechanical properties (i.e., Young’s modulus, flexural stress, and fracture strain) (*p* < 0.0001). The interaction between protein content and nitrogen gas injection pressure on the aforementioned extrudates’ mechanical properties was also significant (*p* < 0.0001).

The Young’s modulus and flexural stress for the conventionally extruded samples ranged from 1.22 to 5.13 MPa and 4.04 to 12.24 MPa, respectively. Similarly, wheat flour extrudates were reported to have Young’s modulus and flexural stress values ranging from 0.02 to 1.3 MPa and 0.4 to 19 MPa and MPa, respectively [12]. Young’s modulus and flexural stress are found to be strongly and positively correlated (r = 0.96, r < 0.0001), indicating stiffer extrudates (i.e., with higher Young’s modulus) tend to withstand greater stress before bending failure.

For conventionally extruded samples, the 0% protein extrudate had the lowest Young’s modulus and flexural stress, while the highest fracture strain indicated the lowest stiffness and strength in bending as well as the highest extensibility for this sample [45]. The addition of protein at a 10% level resulted in the highest Young’s modulus and the lowest fracture strain and, therefore, the most brittle sample among all the conventionally extruded samples. This might be associated with the reduced REI (Figure 2). As evidence, REI and fracture strain are found to be significantly and positively correlated (r = 0.98, *p* < 0.0001), while REI and Young’s modulus are moderately and negatively correlated. Last but not least, the 50% protein extrudates had both the highest Young’s modulus and flexural stress.

For nitrogen gas-assisted extrusion, nitrogen gas injection at 300 kPa significantly increased extrudates’ Young’s modulus and flexural stress while significantly reducing extrudates’ fracture strain at 0% protein content. Conversely, at 10% protein content, nitrogen gas injection at both 150 and 300 kPa significantly decreased Young’s modulus compared to the no-gas injection counterpart. Additionally, nitrogen gas injection at 300 kPa significantly improved fracture strain at this protein level. Similar to the 0% protein formula, at 20% protein, nitrogen gas injection at 300 kPa significantly improved Young’s modulus and flexural stress, while nitrogen gas injection at 150 kPa significantly reduced fracture strain. A further increase in nitrogen gas injection pressure from 150 to 300 kPa reduced fracture strain significantly at 20% protein. At 50% protein, nitrogen gas injection at 300 kPa significantly reduced flexural stress when compared to the no-gas injection counterpart.

The correlation analysis indicated that there were multiple significant correlations between the physical and mechanical properties of the extrudates. For instance, extrudate density and flexural stress are strongly and positively correlated (r = 0.83, *p* < 0.0001), as are density and Young’s modulus, albeit to a moderate degree (r = 0.74, *p* < 0.001). Similar positive correlations between extrudate density and both Young’s modulus and flexural stress were also reported for extruded corn starch and whey protein isolate [43]. In addition, porosity is negatively correlated with both Young’s modulus (r = −0.72, *p* < 0.001) and flexural stress (r = −0.83, *p* < 0.0001). Overall, it can be seen that higher extrudate density and lower extrudate porosity correspond to higher stiffness and stress resistance of extrudates.

In terms of the correlations between texture and mechanical properties, extrudate hardness showed only a moderate correlation with fracture strain (r = 0.51, *p* < 0.05) but no significant correlation with other mechanical properties studied. In contrast, crispness was strongly and negatively correlated with both flexural stress (r = −0.86, *p* < 0.0001) and Young’s modulus (r = −0.85, *p* < 0.0001), suggesting that crisper textures are associated with lower stiffness and lower stress resistance, in line with the results reported for extruded wheat flour and cornmeal [46]. Crispness also showed a moderate positive correlation with fracture strain (r = 0.59, *p* < 0.05), while crunchiness exhibited a strong positive correlation with fracture strain (r = 0.87, *p* < 0.0001). Furthermore, crunchiness had moderate and weak correlations with Young’s modulus (r = 0.50, *p* < 0.05) and flexural stress (r = 0.49, *p* < 0.05), respectively.

## 4. Conclusions

The effects of nitrogen gas injection at the pressures studied were a function of protein content in the feed material. The radial expansion of extrudates showed a significant increase with nitrogen gas injection at 10 and 40% protein, whereas a significant decrease was observed in extrudates with 0 and 20% protein content. Nitrogen gas injection at 150 kPa significantly reduced extrudate density for formulations with 10 and 30% protein. Meanwhile, at 300 kPa, gas injection significantly lowered extrudate density for the 50% protein formulation but significantly increased extrudate density for the 20% protein formulation.

Additionally, some improvements in texture, such as decreases in hardness and increases in crispness, were found with nitrogen gas injection, particularly at the 50% protein incorporation level and 300 kPa gas injection pressure. This study showed the promising potential of nitrogen gas-assisted extrusion in improving the physical appeal of a novel healthy snack at high protein levels (i.e., up to 50%). In the future, a more comprehensive study is required to better understand the links among protein content, nitrogen gas injection, and expansion dynamics (e.g., extrudate growth, shrinkage, and solidification). Future studies will focus on a wider range of extrusion processing conditions. In addition, while this study comprehensively analyzed the texture properties of snacks using a texture analyzer, future research should consider incorporating sensory tests to assess consumer perception, as individual preferences and sensory experiences can vary significantly. Finally, this study exclusively focused on the effect of protein level from one source, leaving room for further investigation into the interaction among nitrogen gas injection in a more complicated food system with various ingredients (e.g., proteins and fibers).

## Figures and Tables

**Table 1:** Effects of feed protein content and nitrogen gas injection pressure on extrusion torque, die pressure, and specific mechanical energy (SME) input during extrusion ^1^.

Feed Protein Content (%, d.b.)	Nitrogen Gas Pressure (kPa)	Die Pressure (kPa)	Torque (%)	SME (Wh/kg)
0	0	1380 ± 20 c	57.4 ± 0.5 cde	265.0 ± 2.1 cde
	150	1260 ± 20 cde	58.3 ± 0.2 cde	269.2 ± 0.9 cde
	300	870 ± 10 hi	59.4 ± 0.2 c	274.2 ± 1.1 c
10	0	1060 ± 60 fg	59.0 ± 0.2 cde	272.3 ± 1.0 cde
	150	880 ± 20 hi	62.2 ±0.5 b	286.9 ± 2.5 b
	300	600 ± 30 j	62.9 ± 0.6 ab	290.4 ± 2.9 ab
20	0	1140 ± 40 def	59.0 ± 0.5 cde	272.3 ± 2.2 cde
	150	1060 ± 20 fg	62.3 ± 0.4 b	287.7 ± 2.0 b
	300	930 ± 10 ghi	62.0 ± 0.4 b	286.2 ± 2.0 b
30	0	920 ± 20 ghi	59.2 ± 0.4 cd	273.1 ± 1.7 cd
	150	820 ± 10 i	56.9 ± 0.4 e	262.7 ±1.7 e
	300	910 ± 10 ghi	58.1 ± 0.4 cde	268.1 ± 1.9 cde
40	0	1300 ± 30 cd	57.2 ± 0.4 de	263.8 ± 1.9 de
	150	1040 ± 20 fgh	59.2 ± 0.4 cd	273.1 ± 1.9 cd
	300	1110 ± 20 ef	58.6 ± 0.2 cde	270.4 ± 0.9 cde
50	0	2230 ± 60 a	51.1 ± 0.6 f	235.8 ± 2.8 f
	150	2410 ± 70 a	52.0 ± 0.5 f	240.0 ± 2.3 f
	300	1960 ± 50 b	65.1 ± 0.6 a	300.4 ± 2.9 a

^1^ Values are presented as mean ± standard error (*n* = 12). For each column, different letters represent statistically significant differences among treatments (*p* < 0.05).

**Table 2:** Two-way ANOVA results for the effects of protein content and nitrogen gas injection pressure on extrusion parameters and extrudate physical quality parameters.

Factors	*p* Value					
	Die Pressure, Torque, SME ^1^, REI ^2^, Extrudate Density	Hardness, Crispness	Crunchiness	Normalized Crispness	Normalized Crunchiness	Young’s Modulus, Flexural Stress, and Fracture Strain
Protein	<0.0001	<0.0001	<0.0001	<0.0001	<0.0001	<0.0001
Nitrogen gas injection pressure	<0.0001	0.0003	<0.0001	0.72	0.03	<0.0001
Protein × Nitrogen gas injection pressure	<0.0001	<0.0001	<0.0001	<0.0001	0.24	<0.0001

^1^ SME: specific mechanical energy ^2^ REI: radial expansion index.

**Table 3:** Pearson’s correlation coefficients (r) for the relationships between processing parameters during extrusion (conventional only) and the physical properties of extrudates.

Variable 1	Variable 2	Pearson’s r	*p*-Value
Motor torque (SME) ^1^	Die pressure	0.99 **^,2^	<0.001
	Porosity	0.98 **	<0.001
	Density	0.96 **	<0.01
	Crispness	0.91 **	<0.05
Die pressure	Fracture strain	0.99 **	<0.001
	Density	0.98 **	<0.001
	Porosity	−0.98 **	<0.001
	Crispness	0.90 **	<0.05
	Young’s modulus	−0.86 **	<0.05
Radial expansion index	Crunchiness	0.95 **	<0.01
	Hardness	0.90 **	<0.05
	Normalized crunchiness	0.85 **	<0.05
Density	Porosity	−0.99 **	<0.001
	Crispness	−0.93 **	<0.01
	Normalized crispness	0.89 **	<0.05
Hardness	Crunchiness	0.94 **	<0.01
	Fracture strain	0.92 **	<0.01
	Normalized crunchiness	0.90 **	<0.05
Crispness	Porosity	0.94 **	<0.01
	Young’s modulus	−0.85 **	<0.05
Crunchiness	Fracture strain	0.97 **	<0.01
	Normalized crunchiness	0.96 **	<0.01
Normalized crunchiness	Fracture strain	0.89 **	<0.05
Young’s modulus	Flexural stress	0.94 **	<0.001
	Fracture strain	−0.86 **	<0.05

^1^ Torque and specific mechanical energy (SME) are considered the same as they have a correlation coefficient of 1. ^2^ Correlation is considered strong (**) when |r| > 0.8, moderate (*) when |r| is between 0.5 and 0.8, and weak when |r| < 0.5 [18].

**Table 4:** Pearson’s correlation coefficients (r) for the relationships between processing parameters during extrusion (conventional and gas-assisted) and the physical properties of extrudates.

Variable 1	Variable 2	Pearson’s r	*p*-Value
Motor torque (SME) ^1^	Die pressure	−0.56 *^,2^	<0.05
Die pressure	Protein content	0.60 *	<0.01
	Porosity	−0.66 *	<0.01
	Density	0.62 *	<0.01
Radial expansion index	Fracture strain	0.98 **	<0.0001
	Crunchiness	0.88 **	<0.0001
	Young’s modulus	−0.75 *	<0.001
	Fracture stress	−0.68 *	<0.01
	Hardness	0.54 *	<0.05
	Crispness	0.56 *	<0.05
Density	Porosity	−0.99 **	<0.001
	Flexural stress	0.83 **	<0.0001
	Crispness	−0.83 **	<0.001
	Young’s modulus	0.74 *	<0.001
	Normalized crispness	−0.73 *	<0.001
Hardness	Normalized crunchiness	0.90 **	<0.0001
	Crunchiness	0.83 **	<0.0001
	Normalized crispness	0.74 *	<0.001
	Fracture strain	0.51 *	<0.05
Crispness	Flexural stress	−0.86 **	<0.0001
	Young’s modulus	−0.85 **	<0.0001
	Porosity	0.79 *	<0.001
	Normalized crispness	0.72 *	<0.001
	Fracture strain	0.59 *	<0.05
Crunchiness	Fracture strain	0.87 **	<0.0001
	Normalized crunchiness	0.80 *	<0.001
	Young’s modulus	−0.50 *	<0.05
	Flexural stress	−0.49	<0.05
Normalized crispness	Flexural stress	−0.52 *	<0.05
	Normalized crunchiness	−0.49	<0.05
Young’s modulus	Flexural stress	0.96 **	<0.0001
	Fracture strain	−0.76 *	<0.001
	Porosity	−0.72 *	<0.001
Flexural stress	Porosity	−0.83 **	<0.0001
	Fracture strain	−0.70 *	<0.01

^1^ Torque and specific mechanical energy (SME) are considered the same as they have a correlation coefficient of 1. ^2^ Correlation is considered strong (**) when |r| > 0.8, moderate (*) when |r| is between 0.5 and 0.8, and weak when |r| < 0.5 [18].

**Table 5:** Effects of feed protein content and nitrogen gas injection pressure on extrusion texture properties. For each column, different letters represent statistically significant differences among treatments (*p* < 0.05).

Feed Protein Content (%, d.b.)	Nitrogen Gas Pressure (kPa)	Hardness (N)	Crispness	Crunchiness	Normalized Crispness (mm^−1^)	Normalized Crunchiness (mm^−1^)	Young’s Modulus (MPa)	Flexural Stress (MPa)	Fracture Strain
0	0	37.231 ± 2.946 a	8.9 ± 0.6 ab	149.365 ± 11.421 a	0.0837 ± 0.060 c–f	12.891 ± 1.071 a	1.219 ± 0.088 h	4.037 ± 0259 f	3.342 ± 0.063 a
	150	29.647 ± 2.095 b	8.2 ± 0.3 ab	115.353 ± 7.584 b	0.775 ± 0.026 ef	10.724 ± 0.590 abc	2.146 ± 0.201 gh	5.977 ± 0.534 ef	2.809 ± 0.071 b
	300	28.464 ± 1.409 b	5.2 ± 0.3 de	69.204 ± 3.032 c–h	0.878 ± 0.054 b–f	11.828 ± 0.607 ab	8.530 ± 0.776 b	11.831 ± 0.958 bc	1.404 ± 0.022 i
10	0	16.766 ± 0.531 de	8.2 ± 0.3 ab	55.219 ± 2.535 fgh	1.192 ± 0.059 a	8.044 ± 0.513 de	5.128 ± 0.495 cde	8.964 ± 0.633 d	1.809 ± 0.056 h
	150	18.086 ± 0.805 de	8.1 ± 0.5 abc	58.230 ± 2.006 d–h	1.105 ± 0.068 abc	7.917 ± 0.324 de	3.801 ± 0.154 efg	7.210 ± 0.251 de	1.906 ± 0.023 gh
	300	16.875 ± 0.741 de	8.5 ± 0.4 ab	56.501 ± 2.501 e–h	1.077 ± 0.051 a–d	7.098 ± 0.309 e	3.188 ± 0.251 fg	6.640 ± 0.415 def	2.139 ± 0.065 ef
20	0	20.980 ± 0.701 cde	9.8 ± 0.4 a	75.249 ± 2.746 c–f	1.069 ± 0.039 a–e	8.196 ± 0.321 cde	3.311 ± 0.297 fg	7.881 ± 0.505 de	2.445 ± 0.059 c
	150	15.517 ± 0.612 e	8.2 ± 0.4 ab	50.788 ± 1.248 gh	1.154 ± 0.036 ab	7.190 ± 0.239 de	4.403 ± 0.299 def	8.423 ± 0.483 de	1.937 ± 0.037 fgh
	300	22.154 ± 0.678 cd	4.1 ± 0.1 e	47.469 ± 1.277 h	0.731 ± 0.019 f	8.540 ± 0.242 cde	14.993 ± 0.578 a	20.188 ± 0.711 a	1.350 ± 0.013 i
30	0	21.379 ± 0.956 cd	9.8 ± 0.5 a	82.004 ± 3.930 c	1.053 ± 0.075 a–e	9.717 ± 0.520 bcd	3.084 ± 0.188 fg	7.380 ± 0.409 de	2.410 ± 0.040 cd
	150	19.665 ± 0.804 de	10.2 ± 0.5 a	75.920 ± 3.878 c–f	1.162 ± 0.05 0 ab	8.811 ± 0.480 cde	2.460 ± 0.178 gh	5.914 ± 0.354 ef	2.454 ± 0.061 c
	300	21.186 ± 0.517 cde	9.8 ± 0.5 a	79.844 ± 2.824 cd	1.091 ± 0.058 abc	9.131 ± 0.313 cde	2.555 ± 0.135 gh	6.177 ± 0.255 ef	2.445 ± 0.048 c
40	0	18.805 ± 0.862 de	9.0 ± 0.6 ab	70.374 ± 3.976 c–g	1.209 ± 0.080 a	9.319 ± 0.676 b–e	3.736 ± 0.245 efg	7.569 ± 0.460 de	2.038 ± 0.027 efg
	150	18.410 ± 0.980 de	9.5 ± 0.7 a	70.053 ± 3.035 c–g	1.215 ± 0.083 a	8.348 ± 0.334 cde	3.693 ± 0.231 efg	7.821 ± 0.442 de	2.130 ± 0.022 efg
	300	19.226 ± 0.786 de	9.8 ± 0.7 a	72.334 ± 2.619 c–g	1.210 ± 0.082 a	9.208 ± 0.346 cde	3.530 ± 0.308 efg	7.519 ± 0.616 de	2.144 ± 0.022 ef
50	0	26.007 ± 1.075 bc	5.8 ± 0.4 cde	72.607 ± 3.333 c–g	0.721 ± 0.054 f	8.964 ± 0.485 cde	5.548 ± 0.342 cd	12.239 ± 0.691 b	2.216 ± 0.017 de
	150	25.511 ± 0.575 bc	6.6 ± 0.5 bcd	77.873 ± 5.769 cde	0.793 ± 0.053 def	9.437 ± 0.635 b–e	6.174 ± 0.390 c	13.479 ± 0.806 b	2.195 ± 0.026 de
	300	19.135 ± 0.505 de	8.1 ± 0.5 abc	66.397 ± 3.001 c–h	1.059 ± 0.065 a–e	8.646 ± 0.360 cde	4.536 ± 0.269 cdef	9.175 ± 0.473 cd	2.039 ± 0.034 efg

Values are presented as mean ± standard error (*n* = 9 for columns 3–7 and *n* = 15 for columns 8–10).

## Data Availability

The original contributions presented in the study are included in the article/Supplementary Materials, further inquiries can be directed to the corresponding author.

## Supplementary Materials

The following supporting information can be downloaded at https://www.mdpi.com/article/10.3390/foods13152411/s1, Figure S1: Digital photo of key extrudates produced at varying protein content and nitrogen gas injection pressure. The scale bar at the bottom right represents 10 mm length.
