# Supplementary material for: Nitrogen Gas-Assisted Extrusion for Improving the Physical Quality of Pea Protein-Enriched Corn Puffs with a Wide Range of Protein Contents
Source: Foods. 2024 Jul 30;13(15):2411. doi: 10.3390/foods13152411 (PMC11311776; doi:10.3390/foods13152411)
Supplement: Supplementary file 1 [file foods-13-02411-s001.zip › foods-3076317-supplementary.pdf]

Supplementary material

For

Nitrogen Gas-Assisted Extrusion for Improving the Physical Quality of Pea Protein-Enriched Corn Puffs with a Wide Range of Protein Contents

Siwen Luo [1], Jitendra Paliwal [2] and Filiz Koksel [1,*]

[1] Department of Food and Human Nutritional Sciences, University of Manitoba, Richardson Centre for Food Technology and Research, 196 Innovation Drive, Winnipeg, MB R3T 2N2, Canada; luos345@myumanitoba.ca
[2] Department of Biosystems Engineering, University of Manitoba, E2-376, EITC, 75A Chancellor's Circle, Winnipeg, MB R3T 2N2, Canada; j.paliwal@umanitoba.ca
* Correspondence: filiz.koksel@umanitoba.ca

Outline:

This supplementary material contains a digital photo (Figure S1) illustrating the appearance of extrudates produced by varying feed protein contents and nitrogen gas injection pressures.

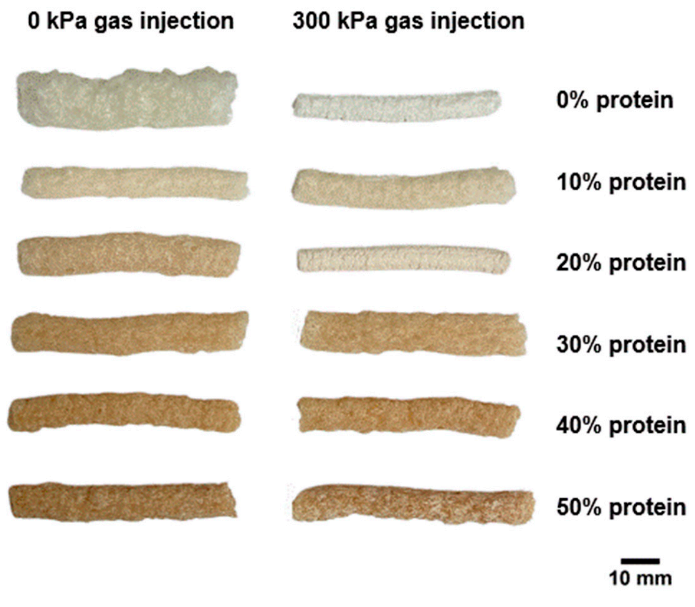

Figure S1. Digital photo of key extrudates produced at varying protein content and nitrogen gas injection pressure. The scale bar at the right bottom represents 10 mm length.
